# NBBC: a non-B DNA burden explorer in cancer

**DOI:** 10.1093/nar/gkad379

**Published:** 2023-05-24

**Authors:** Qi Xu, Jeanne Kowalski

**Affiliations:** Department of Oncology, Dell Medical School, The University of Texas at Austin, Austin, TX 78712, USA; Department of Molecular Biosciences, The University of Texas at Austin, Austin, TX 78712, USA; Department of Oncology, Dell Medical School, The University of Texas at Austin, Austin, TX 78712, USA

## Abstract

Alternate (non-B) DNA-forming structures, such as Z-DNA, G-quadruplex, triplex have demonstrated a potential role in cancer etiology. It has been found that non-B DNA-forming sequences can stimulate genetic instability in human cancer genomes, implicating them in the development of cancer and other genetic diseases. While there exist several non-B prediction tools and databases, they lack the ability to both analyze and visualize non-B data within a cancer context. Herein, we introduce NBBC, a non-B DNA burden explorer in cancer, that offers analyses and visualizations for non-B DNA forming motifs. To do so, we introduce ‘non-B burden’ as a metric to summarize the prevalence of non-B DNA motifs at the gene-, signature- and genomic site-levels. Using our non-B burden metric, we developed two analyses modules within a cancer context to assist in exploring both gene- and motif-level non-B type heterogeneity among gene signatures. NBBC is designed to serve as a new analysis and visualization platform for the exploration of non-B DNA, guided by non-B burden as a novel marker.

## Graphical Abstract

**Graphical Abstract:**
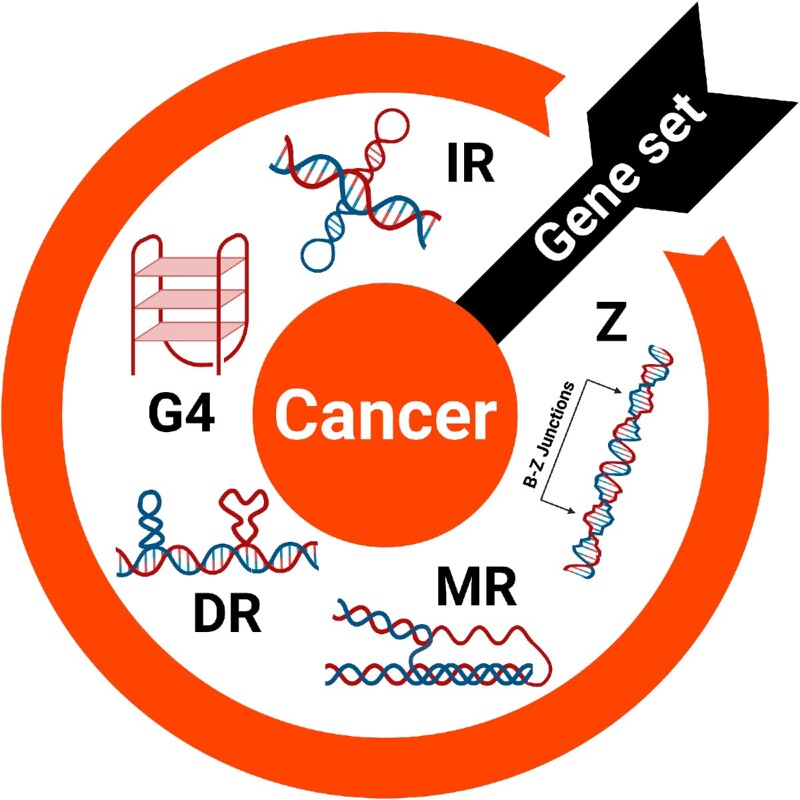

## INTRODUCTION

Non-canonical (non-B) DNA refers to DNA structures that differ from the canonical B-DNA double helix structure. These non-B DNA structures include G-quadruplex, cruciform, triplex, slipped structures, left-handed Z-DNA and others (1–3). It has been reported that approximately 13% of the human genome can form into non-B DNA structures and this approximation can also vary depending on multiple factors including cellular types, cell processes and other factors.

Non-B DNA-forming sequences in genomes affect DNA replication and transcription. It has been found that non-B DNA-forming sequences can stimulate genetic instability in human cancer genomes, implicating them in the development of cancer (1). However, the mechanisms by which non-B DNA structures contribute to cancer are not yet fully understood. It is known that these structures can perturb the normal processes of central dogma. For example, DNA triplex and G-quadruplex structure formation may regulate the expression of cancer-related genes via these non-canonical structures (2). Correlation analyses of DNA structure and gene expression with mutation loads complement and extend more traditional approaches to elucidate the mechanisms underlying cancer development (4). Additionally, genomic features such as histone epigenetic marks and replication time domains have been shown to be predictive of the variation in distribution of somatic mutations in non-B DNA motifs (5). Increased mutability has been identified within non-B DNA motifs. Z-DNA has been demonstrated to act as a positive regulator of gene expression and G-quadruplexes shown to influence promoter activities (6). Cancer mutational burden is shaped by G4 DNA, replication time domains, and other factors (4). Non-canonical DNA structures have been implicated as drivers of genome evolution (3).

While there exist several non-B prediction databases (7,8), they lack the ability to both analyze and visualize non-B data within the context of cancer gene signature sets. Herein, we introduce NBBC, a non-B DNA burden explorer in cancer to address this gap. For this purpose, we introduce ‘non-B burden’ as a metric to summarize the prevalence of non-B DNA motifs. NBBC includes two main analyses modules: ‘gene screen’ and ‘motif screen’ module. The ‘gene screen’ layer serves to conduct non-B burden computations and offers normalizations that enable comparisons across genes or non-B structures. It provides visualizations for descriptive analysis of burden values, burden distribution, and burden-based gene clustering. The ‘motif screen’ layer is focused on motif exploration and is designed to define motifs with similar features, in terms of length and %guanine content. For input, NBBC takes genes symbols, gene signatures, genomic regions, either as a single query or in batch. It outputs DNA burdens either by non-B types or in total at gene level or at group level.

NBBC serves as a valuable resource for researchers investigating the role of non-B DNA structures in cancer and other genetic diseases. By offering an accessible platform for analyzing and visualizing non-B DNA motifs within a cancer context, NBBC enables the exploration of non-B structures by a wide, non-bioinformatic user base.

## MATERIALS AND METHODS

### The web application architecture and cloud deployment

The NBBC web application was created using Shiny R and features an intuitive interface that has been enhanced with HTML widgets, JavaScript, and Cascading Style Sheets (CSS). The application is hosted on Amazon Web Services (AWS) using the AWS Fargate cloud architecture, which utilizes containerization and a serverless approach. To ensure optimal scalability and user experience, an AWS Elastic Load Balancer (ELB) is employed as a traffic controller.

### Data source and data pre-processing

The non-B DNA forming motif data was obtained from Non-B DB 2.0 database with the hg19 build (7). An update to correct the A-Phased repeat motifs data was received from Frederick National Laboratory for Cancer Research (personal communication). There are motifs of 7 non-B structures including: A-phased repeats (APR, *n* = 2386 motifs), G-quadruplex motifs (G4, *n* = 361 232 motifs), Z-DNA motifs (*n* = 404 192 motifs), inverted repeats (IR, *n* = 5 771 570 motifs), mirror repeats (MR, *n* = 1 378 864 motifs), direct repeats (DR, *n* = 1 113 354 motifs), and short tandem repeats (STR, *n* = 2 826 360 motifs). A subset of MR and IR motifs are further delineated within the application to represent triplex forming motifs (Triplex-MR, *n* = 412 028 motifs) and cruciform forming motifs (Cruciform-IR, *n* = 147 152 motifs) respectively. For input, NBBC offers several built-in cancer related gene sets for quick queries, including cancer hallmark gene signatures from MSigDB databases (9), DNA damage repair and response gene signatures from Lange *et al.* (10). Additionally, cancer cell line molecular signatures are extracted from Genomics of Drug Sensitivity in Cancer (GDSC) database (11).

### Non-B burden calculation and normalization

The non-B burden is introduced to evaluate and compare the prevalence of non-B structures. It is estimated by counting the number of non-B forming regions of each non-B type in each gene. To compare the non-B burdens across different genes or different non-B types, normalization metrics are applied and provided in NBBC. The various non-B burden metrics included are: raw motif counts (without normalization), normalization by region length, normalization by motif library size, and normalization by both length and library size. The default unit of non-B burden in NBBC is CPKM, counts per kilobase per million. This is used to normalize the non-B motif prevalence (counts) by the length of query regions (per kilobase, 10^3^) and by the library sizes of non-B motifs (per million, 10^6^). Normalization allows the comparison of non-B burden across regions (such as difference gene region) and across difference non-B types.

In specific, region and motif library normalized non-B burden is defined as:


(1) $$\begin{eqnarray*}\frac{{Counts\ of\ nonB\ motifs\ overlapped\ with\ query\ regions \times {{10}}^3\ \times {{10}}^6}}{{Total\ nonB\ library\ size \times Total\ query\ region\ length\ }}\nonumber\\ \end{eqnarray*}$$

Here, 10^3^ normalizes for query region length and 10^6^ for non-B library size factor.

### Non-B burden visualization

NBBC offers various visualizations for non-B burden quantification, facilitating the analysis and comparison of individual or multiple genes in terms of their non-B burden composition. A bar plot is used to visualize the total non-B burden. A stacked bar plot and a bubble plot allow users to see the non-B burden by gene and type. A burden clustering function is available with the a heatmap visualization. A distribution plot enables users to select genes with high and low burden from statistical intervals. Major visualizations are produced using the R packages ggplot2 (12) and plotly, with the interactive features provided by R Shiny (13). Heatmaps are visualized using the ComplexHeatmap package (14).

### Non-B motif clustering

The motif screen performs sequence-level motif clustering for high-quality non-B motif detection. For example, the length and guanine content (%G) are two major factors in deciding motif quality for non-B forming. We employ unsupervised clustering to define motifs with similar length and %G. The app supports multiple features for clustering including length, guanine, adenine compositions in the non-B motif sequences. The K-means clustering method is applied for non-B forming motifs clustering. The R packages ggrepel and ggalt are used to enhance visualization.

### Flank region extraction of non-B forming sequence

The flank region extraction feature allows users to obtain the non-B forming regions, including additional flank sequences on both ends. This functionality facilitates further investigation beyond the scope of the application. The bedtools utilities (15) are employed to accomplish this extraction process.

## RESULTS

### Overall design of NBBC

**Figure 1.**
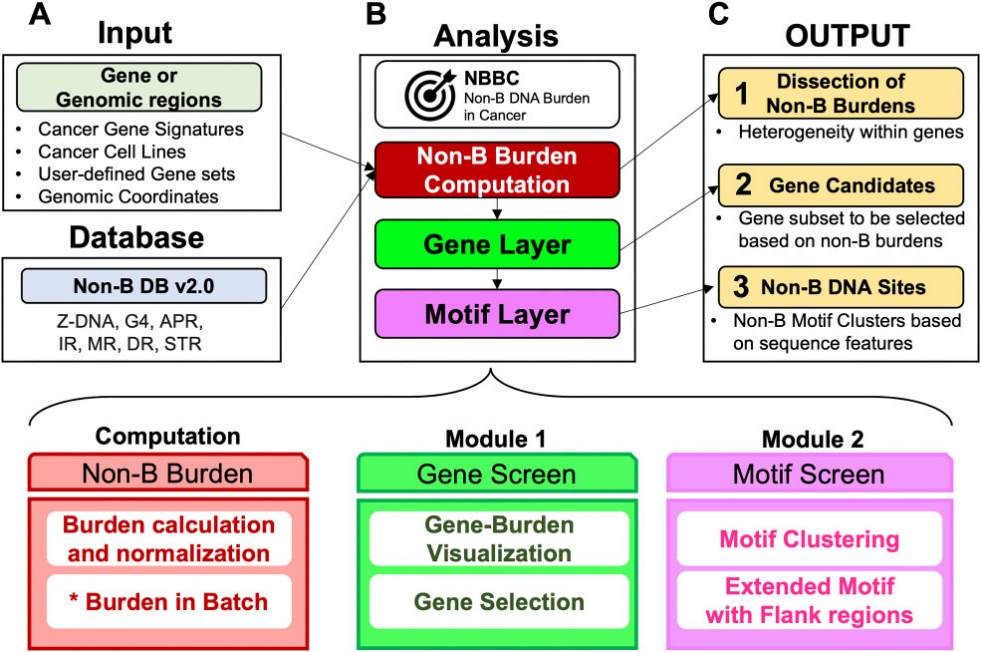
The overall design of NBBC. (**A**) The input includes genomic regions in query and non-B types. (**B**) The first module is ‘Gene Screen’. The gene layer analyses non-B burden for input gene query. And the second module is ‘Motif screen’ that performs sequence clustering. (**C**) The output includes the dissection of burden in the query regions, genes with high burdens and non-B DNA sites with user-desired features.

The NBBC web server consists of three core functional modules. The overall design of NBBC is summarized in Figure 1. The first module is ‘gene screen’. This layer offers several computation and analysis options based on non-B burden for input query genes or regions. In terms of computation, this module derives non-B burden calculation in user-selected units to examine non-B burden composition for a query (on multiple gene levels) alongside several normalization options to facilitate non-B burden comparisons among genes and/or non-B structures. Several descriptive analyses are offered in the gene screen module with visualizations for exploring non-B burden values, distribution, and clustering at the gene-level. The second module offered in NBBC is ‘motif screen’ in which users are able to undertake a more focused exploration of non-B motifs. Corresponding to the query of interest for analyses, users are able to perform clustering on any combination of motif-associated features: length, guanine content (%G), and adenine content (%A). This capability allows users to conduct a more focused search for motifs with characteristics of interest in the context of their research.

### Input format options

**Figure 2.**
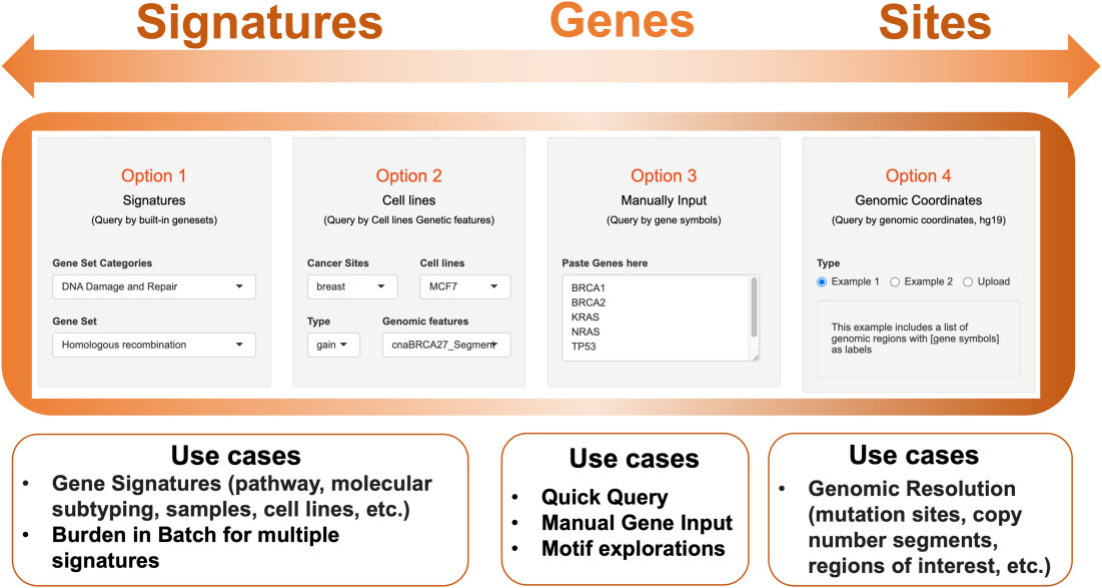
Introduction to input options. NBBC supports multi-level burden query and the current version provides four options at three levels for different goals and user circumstances. (**A**) Signature-level input. The input includes popular cancer signatures, cell line molecular signatures or user-defined signatures. (**B**) Gene-level input. The typical use case is a quick single gene search by manual input and motif exploration with the query gene. (**C**) Site-level input. It applies to burden queries at the high genomic resolution, such as cancer-specific mutation sites or regions with copy number alterations.

The NBBC app accepts input from three different levels: gene- signature-, and site-level (Figure 2). The web server provides four options with which to satisfy users’ input requirements. The first option includes built-in cancer-related signature gene sets from which the user can select that include DNA damage repair and response gene pathways, cancer hallmark gene sets, oncogenes etc. A second built-in input option for user selection covers cancer cell line-specific molecular features that include mutations and copy number alterations (11). The third input option allows users to manually input a single or several genes through the web interfaces when a quick gene query is of interest. The fourth input option allows users to upload a set of genomic coordinates representing genomic regions of interest, such as mutation sites, in which non-B burden is placed in the context of mutation-localized non-B burden. With these multiple options, the NBBC app covers non-B calculation at multiple levels and genomic resolution, from precise mutation sites to broad gene signatures. Additionally, NBBC offers a ‘burden in batch’ option that defines non-B burden types for a set of signatures (e.g. molecular subtyping, samples, patient-derived models, etc.) to further explore the use of this potential marker in downstream analyses and experiments.

### Output from gene screen: gene exploration of non-B burden for focused selection

**Figure 3.**
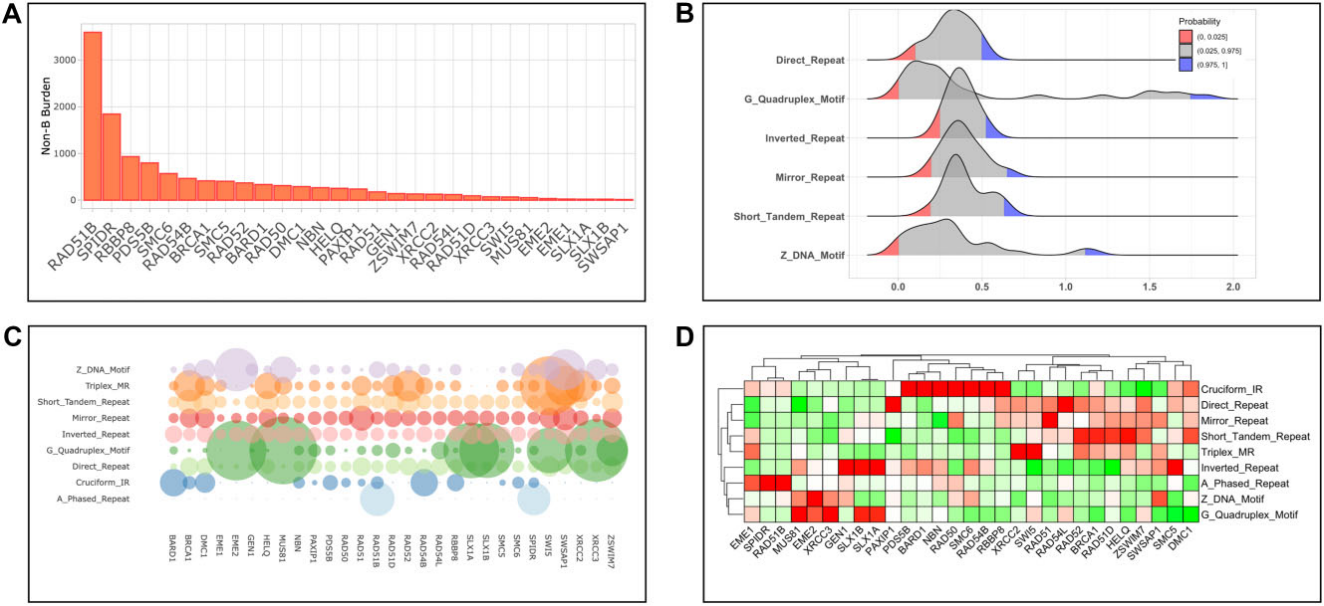
Gene screen layer module to explore non-B burden. The gene layer analyzes non-B burdens and provides several visualizations for descriptive analysis of burden values, burden distribution, and burden-based gene clustering. (**A**) A stacked bar plot is used to visualize the total non-B burden. (**B**) The distribution of non-B burdens by non-B structure motif types. (**C**) A bubble plot allows users to observe the non-B burden by gene and type. (**D**) A burden clustering function is also available in the heatmap format.

The output of initial query of non-B burden is a matrix formed by a list of genes and a list of non-B types. The data in the matrix represent the non-B burdens calculated by the web server and can be scaled with multiple types of normalizations offered in the app. The goal of gene layer in NBBC is to conduct gene-level analyses of non-B representation that could prove helpful in focusing upon a single or subset of genes of interest for hypothesis generation. To address this goal, we visually dissect non-B burden into (a) burden distribution of each non-B type among the query genes; (b) total (cumulative) non-B burden for each gene in query; (c) composition of non-B burden representations at the gene level and (d) heatmap clustering of non-B burden among non-B types and genes (Figure 3).

### Output from motif screen: motif feature exploration of genes from gene screen for sequence focused selection

**Figure 4.**
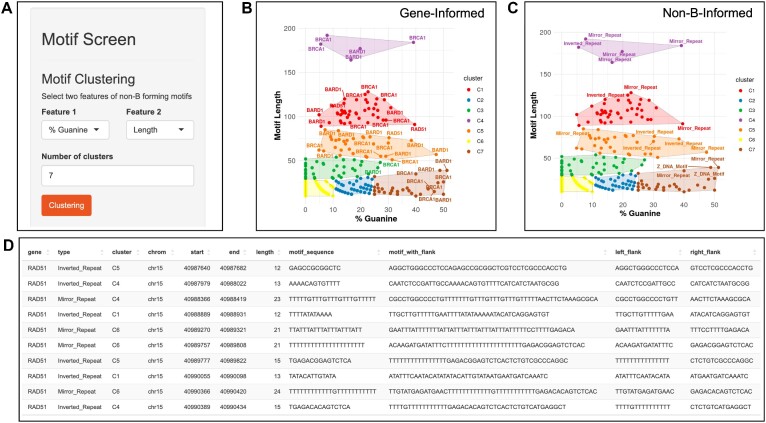
Motif screen layer module to explore viable non-B forming sequences. We use this module to further select high-quality motifs that are more likely to form non-B structures in the interested genes and to provide the specific sequences that can be used for wet lab experiments. (**A**) The interface for motif clustering. Users can choose two of pre-summarized motif sequence features to perform 2D clustering. (**B**, **C**) Clusters of motifs based on sequence features. In the example, motifs with high Guanine content (G%) and decent lengths are highlight as good candidates for user to focus on. Two visualizations are presented labeling gene names (left, gene-informed) or non-B types (right, non-B informed). (**D**) For cases where users are interested in flank regions of a motifs, the app helps output left- and right-flank regions for motifs based on user-input length. The step is achieved with real-time reference genome query.

The motif layer is designed for non-B motif-level exploration and selection of motifs from the gene screen analyses for insights on their heterogeneity with respect to user-selected sequence features: length, %G and %A. For this purpose, the motif screen module offers motif sequence-level unsupervised clustering of features. For example, length and %G can be two major factors to consider when exploring motif selection from mirror repeats (Figure 4A). Clustering of non-B forming sequences based on chosen sequence features can be viewed at both the gene-level (gene-informed) and non-B structure level (non-B informed). The clustering outcomes are represented using two visualizations, with each motif labeled by gene symbols and non-B types (Figure 4B, C). Within each visualization, users can select individual points or encircle a region on the graph to identify non-B motif sequences of interest. The chosen data points are then displayed in a table format, where users have the option to download or to include flank regions of motif sequences for additional downstream exploration (Figure 4D).

## CASE STUDIES

NBBC supports multiple levels of gene and gene region queries to satisfy potential use cases. The basic input of NBBC includes either gene symbols (gene-or signature-level) or defined genomic regions (site-level), singly or in batch. At the gene-level, users can choose to input a single gene or gene signature. In case 1, we demonstrate the fundamental query of non-B burden for a single gene and corresponding burden composition among non-B types (Supplementary Figure S1A). In case 2, we demonstrate user query of a gene signature for non-B burden heterogeneity analyses. (Supplementary Figure S1B). At the site-level, users can upload multiple genomic regions, such as at mutation sites, for non-B burden computation. To accomplish this task for multiple regions from multiple samples, the module, ‘Burden in Batch’ was designed (Supplementary Figure S1C). This option is especially useful for generalized queries based on a set of signatures, such as from model systems and molecular subtypes. We demonstrate in case 3 the application and advantage from use of this option.

### Case 1: gene-level non-B burden (single gene)

**Use cases:** The goal of this use case is to demonstrate the fundamental query of a single gene for non-B burden analyses.

**Example:** Non-B DNA motifs affect mutation rate and facilitate genome instability (3). The *BRCA1* gene is one of the genes most commonly affected in hereditary breast and ovarian cancer (16). *BRCA1* is a key DNA-repair protein, and its functional loss leaves some cells highly vulnerable to DNA damage, including damage that triggers cancer (17). Triple negative/basal-like tumors often accompany *BRCA1* gene mutations and are aggressive with a poorer prognosis. From NBBC, we observe *BRCA1* to have the highest burden (burden CPKM = 0.84) from the triplex-forming sequences (H-DNA) and STR is the second highest burden source (burden CPKM = 0.65) (Supplementary Figure S2B). H-DNA is a triple helix secondary structure formed by homopurine-homopyrimidine sequences with a minimum length of 12 nucleotides (6). The G-content and length of DNA can affect the formation of non-B DNA structures, including H-DNA motifs. To further check the quality of motifs by looking into their composition, we use ‘motif screen’ module to find those with both high %G and long motif lengths. Our cluster analyses of motif features revealed two triplex forming mirror repeat motifs residing on Chromosome 17 with relatively long length and high %G among all forming motifs (Supplementary Figure S2C). The two sequences are: ‘AAAGAGAGAGAGAGAGCAAGAGAGAGAGAGAGAAA’ (length = 35, G% = 40%) and ‘TGTGTGTGCGCGTGTGCGTGTGTGT’ (length = 25, G% = 48%). The app can also output flank regions of the motif regions. For instance, the sequence of the first motif with flanking regions from both left and right sides will be ‘TCTTGGGAAAAAAAA—AAAGAGAGAGAGAGAGCAAGAGAGAGAGAGAGAAA—GACACCCCAGTGAAG’ (the left flank: TCTTGGGAAAAAAAA; the right flank: GACACCCCAGTGAAG).

### Case 2: signature-level analysis of non-B burden heterogeneity (multiple genes)

**Use cases:** The goal of this use case is to demonstrate the application of a gene signature query for performing non-B burden analyses. As opposed to a single gene query, a multiple gene query involves comparisons not only across non-B types but also across genes. Therefore, proper normalizations (gene length and non-B library size) of burdens are applied. For multiple signatures, our burden in batch module may be used to output non-B burdens for multiple gene lists.

**Example:** Poly (ADP-ribose) polymerase inhibitors (PARPi) have shown efficacy in treating cancers with HR deficiencies, including those with mutations in the *BRCA1* and *BRCA2* genes, which are critical for homologous recombination (HR) repair. Non-B DNA structures are known to contribute to genetic instability and evolution, and they are recognized by DNA repair pathways, including the HR pathway (18). G4 stabilization can activate the HR pathway, leading to bypass/repair of G4-mediated DNA damage (19). Other non-B DNA structures, such as triplexes, can also interfere with HR repair, and their presence can affect genomic instability (20). We used NBBC to explore the non-B DNA forming structure heterogeneity among 12 genes in the HR pathway: *BRCA1, BRCA2, MRE11A, RAD51, ATM, CDK12, PALB2, CHEK2, RAD51C, RAD51D, BRIP1* and *BARD1*. Using the ‘gene screen’ interface, we derived normalized total (among non-B types) burden for each gene, which resulted in *CHEK2, BRCA2*, and *PALB2* as the top three genes with the highest total non-B burden. According to the dissection of non-B burden by each structure type, we observed that several high burdens appear to result from Triplex-forming MR, Cruciform IR and direct repeats (Supplementary Figure S3B). For the *CHEK2* gene in particular, the main sources of non-B burdens are from Triplex-MR (burden CPKM = 0.8, Cruciform-IR (CPKM = 0.62), and direct repeat (CPKM = 0.57). We next invoked the motif screen module and performed unsupervised clustering using motif length and %G feature. Taking *CHEK2* and *PALB2* for instance, there are three specific motifs associated with direct repeat forming DNA structures with relatively long length and high %G (Supplementary Figure S3C). Upon extracting these specific sequences, it allows further exploration of their potential role in PARPi response.

### Case 3: site-level analysis of non-B burden heterogeneity among multiple mutations and samples (multiple groups of multiple regions)

**Use cases:** The goal of this use case is to demonstrate the ability to explore non-B burden localized to site-level genomic coordinates from multiple genes and samples with the ‘burden in batch’ option.

**Application:** We applied mutation-localized non-B burdens calculation to genome-wide mutation sites from 104 early-stage pancreatic cancer patients with mutation and survival data from TCGA. In other words, 104 groups of genomic mutation regions from 104 samples were used as input for the burden-in-batch calculation (Supplementary Figure S4A). Each group has its own specific mutation sites signature per sample. The mutation sites of each group were overlapped with non-B forming motif regions to calculate the non-B burden within each sample. For each sample, we derived a site-level non-B burden for each non-B DNA structure, resulting in a non-B burden output matrix of 104 (columns, input groups) × 6 (rows, non-B types) (Supplementary Figure S4B). We performed a cluster analysis on these non-B burdens and compared overall survival (OS) between the groups (Supplementary Figure S4C). Among the 104 early-stage pancreatic patients, non-B burden clustering resulted in six patient clusters that differentiated by non-B DNA structure burden, in which IR high burden samples (*n* = 23, median OS = 15 month) significantly differed in OS from DR high burden samples (*n* = 23, median OS = 30 month). The resulting output matrix of burdens on these sample can be used for other downstream analyses including supervised and unsupervised clustering, total burden calculation, association analyses and more depending on research questions.

## DISCUSSION

The web server supports multiple types of input to maximize the experience, applicability and flexibility for a broad user base. The carefully designed visualizations will satisfy users’ needs and will benefit the non-computational biologists to explore the non-B forming DNA sequence and the genomic instability burden associated in cancer gene signatures.

Overall, NBBC serves as a user-friendly web platform to explore non-B DNA sequences, calculate non-B burden for a first-time look at non-B DNA heterogeneity analyses, and to select of motifs by user-input features of interest. The web server makes it possible to provide scientists with a visualized study of non-B forming DNA while offering a novel approach in which to quantitate non-B burden and study its use as a potential marker of genomic instability in cancer.

Recognizing the importance of considering alternative databases, we have included a feature available in the local instance that allows users to include their own non-B library. To this end, a user can replace the current non-B library with their own choice and compare results by running the application with different database sources.

The recently published complete telomere-to-telomere assembly of the human genome (21), which reveals a higher abundance of non-B DNA-forming sequences than previously identified, highlights the relevance of our application tool. The new reference genome, T2T-CHM13, fills up the small portion (8%) of the genome previously left out that does not produce proteins and comprises highly repetitive DNA sequences located within and surrounding the telomeres and centromeres. This update covers a greater extent of repetitive DNA sequences that may offer further insights into non-B structures within the context of cancer. NBBC’s support for user-defined non-B libraries facilitates the application of new databases based on the updated reference genome, thus offering greater flexibility and adaptability.

In the future, we also plan to further develop NBBC by providing additional support for a motif feature focus with interpretation of input sequences. Currently NBBC focuses on the reference genome, which is where the non-B forming sequences are predicted using non-B DB 2.0 database (7). Although an input option for genomic coordinates has been provided, to have an input module that accepts a user-defined sequence will further extend the use case capabilities of NBBC. However, in order to achieve this function, simply adding a sequence input option may not be sufficient. Currently, non-B forming motifs derived from the reference genome have been pre-computed and their use widely accepted as hence, built into the application. For user-defined sequences, among other things, there would need to be a formal exploration of how closely the reference genome resembles that of an input sequence to determine an accuracy level in prediction and represents a future research area.

Additionally, the current workflow of non-B motif clustering is depending on general sequence properties such as sequence length and sequence composition. Considering the complexity of non-B forming sequences with various kinds of repeat pattern, there is an opportunity to extract more features for exploration of non-B structure forming DNA sequences that will benefit the study of genomic instability in cancer.

Despite the growing interest in non-B DNA research, outside of databases with the sole purpose of ‘look-up’ queries for interrogation, there is little to offer for analyses and exploration of non-B DNA motifs alone and within a cancer context. The lack of comparable tools is underscored by the need to first derive a novel metric of non-B DNA burden as we have introduced, and then to use that metric for an analysis of non-B type heterogeneity as we have accomplished with the development of NBBC.

## DATA AVAILABILITY

The NBBC (https://kowalski-labapps.dellmed.utexas.edu/NBBC/) is a freely available web server that does not require a login. A cookie consent form will be notified to users for first-time visits. Documentation for the app is presented in a bootstrap-styled R book accessible under “App Guide” tab in the application.

## Supplementary Material

gkad379_Supplemental_File
