# Supplementary material for: NBBC: a non-B DNA burden explorer in cancer
Source: Nucleic Acids Res. 2023 May 24;51(W1):W357–64. doi: 10.1093/nar/gkad379 (PMC10320089; doi:10.1093/nar/gkad379)
Supplement: gkad379_Supplemental_File [file gkad379_supplemental_file.pdf]

Supplementary

NBBC: A Non-B DNA Burden Explorer in Cancer

Qi Xu[1], Jeanne Kowalski[1*]

[1] Department of Oncology, Dell Medical School, University of Texas at Austin, Austin, TX, 78712

* To whom correspondence should be addressed.
Tel: 512-495-5737; Email: Jeanne.Kowalski@austin.utexas.edu

Figures and Figure legends
(Case studies: Case 1 – 3)

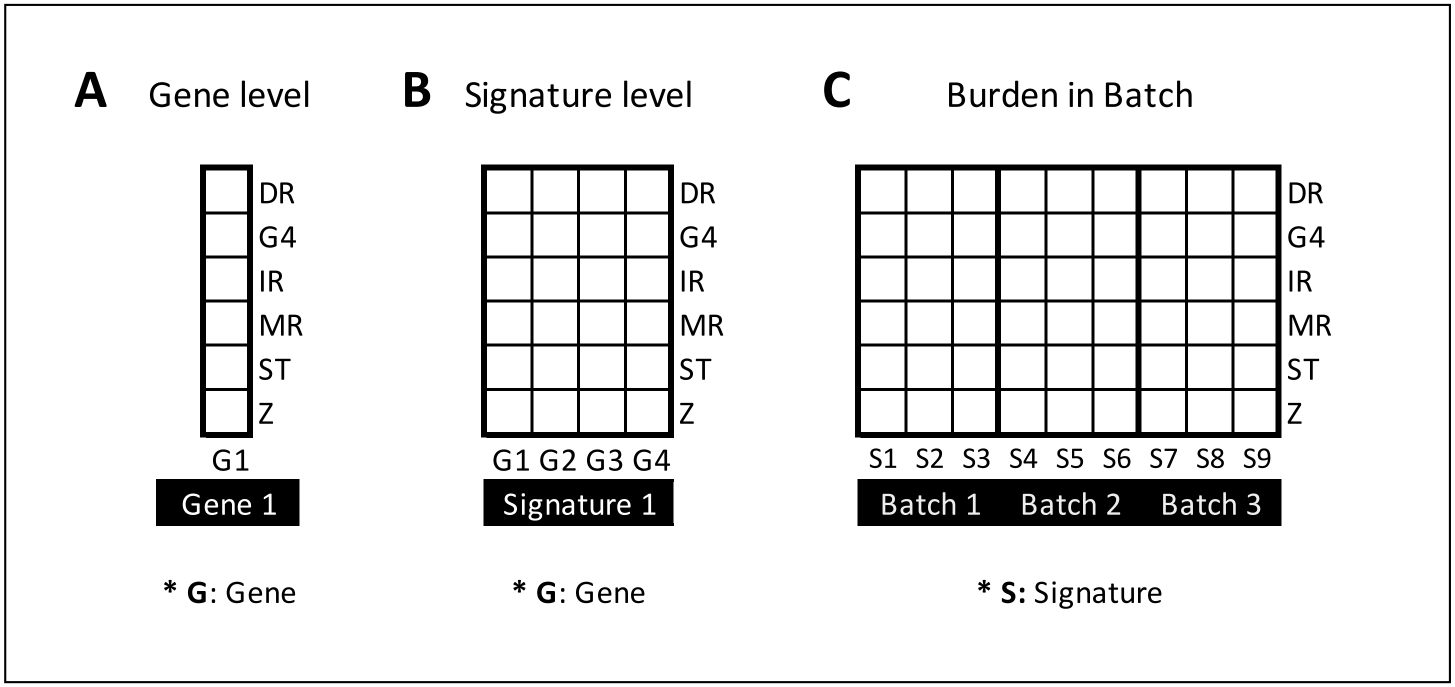

Fig S1. The graphical introduction of input levels for non-B burden calculations. (**A**) A single gene query outputs DNA burdens by non-B types. (**B**) A multiple-gene query outputs DNA burdens by non-B type across genes. (**C**) The "Burden in Batch" enables non-B burden computation by inputting multiple signatures in batch.

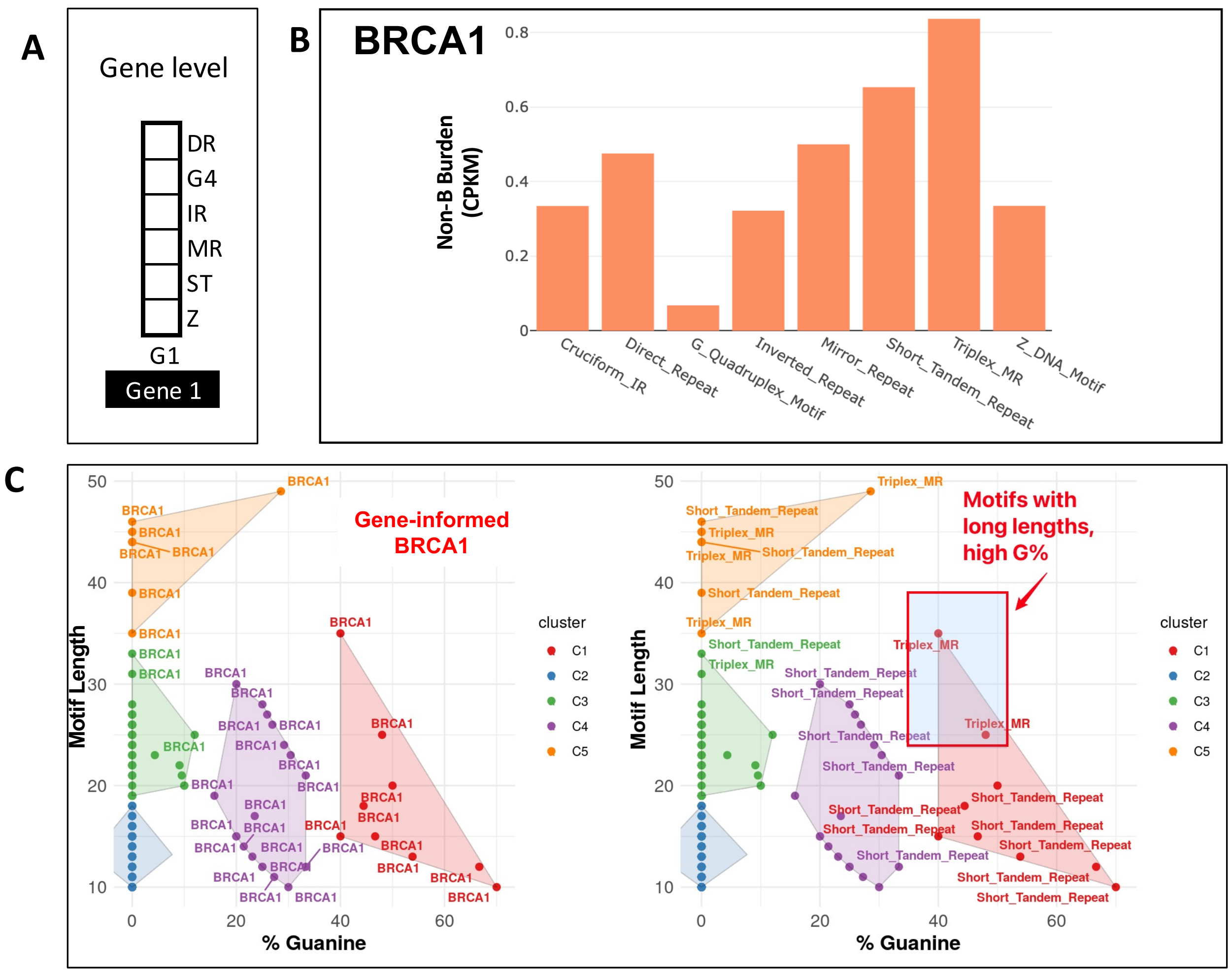

Fig S2. Case 1: Single Gene non-B burdens (Gene-level). Calculation non-B burden and non-b motif screen for single gene query. (**A**) The graphical description of a single gene query. (**B**) Non-B Burden heterogeneity in *BRCA1* by 6 non-B types or subsets (DR, G4, STR, Z-DNA, Triplex-MR, and Cruciform-IR) shown in a bar plot. (**C**) There are 2 mirror repeat motifs found with high G% and long lengths in BRCA1 that may form triplex structures.

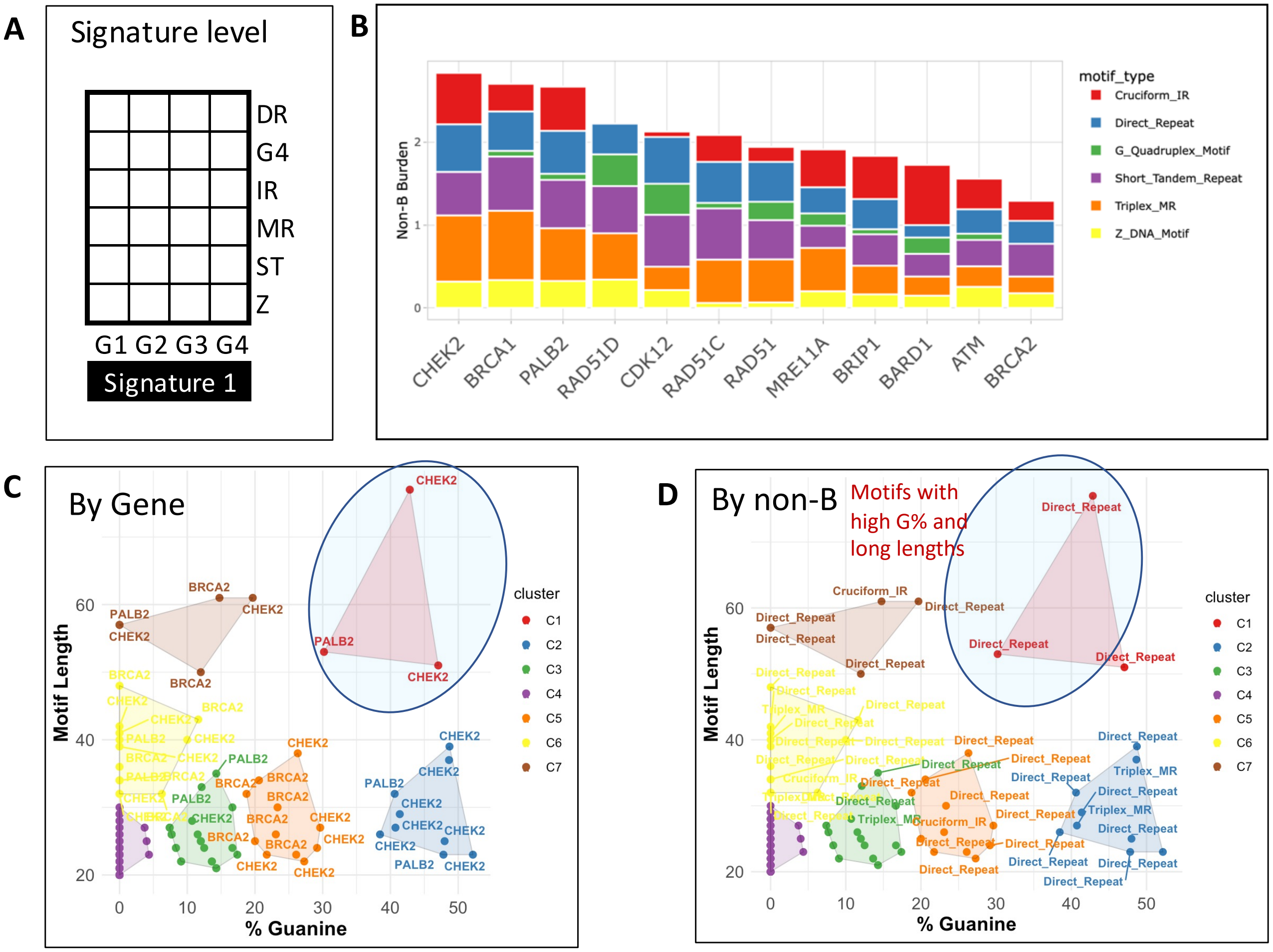

Fig S3. Case 2: Analyses of non-B Burden in genes from homologous repair (HR) pathway. (**A**) Graphical description of multiple gene query. (**B**) The composition of non-B burdens (from STR, G4, DR, Triplex-MR, Cruciform-IR, and Z-DNA) for each of the 11 genes from the HR pathway shown in a stacked bar plot. (C-D) Motif clustering identifies three direct repeats in PALB2 and CHEK2 as having high G% and lengths based on (**C**) the gene-informed and (**D**) non-B informed analyses.

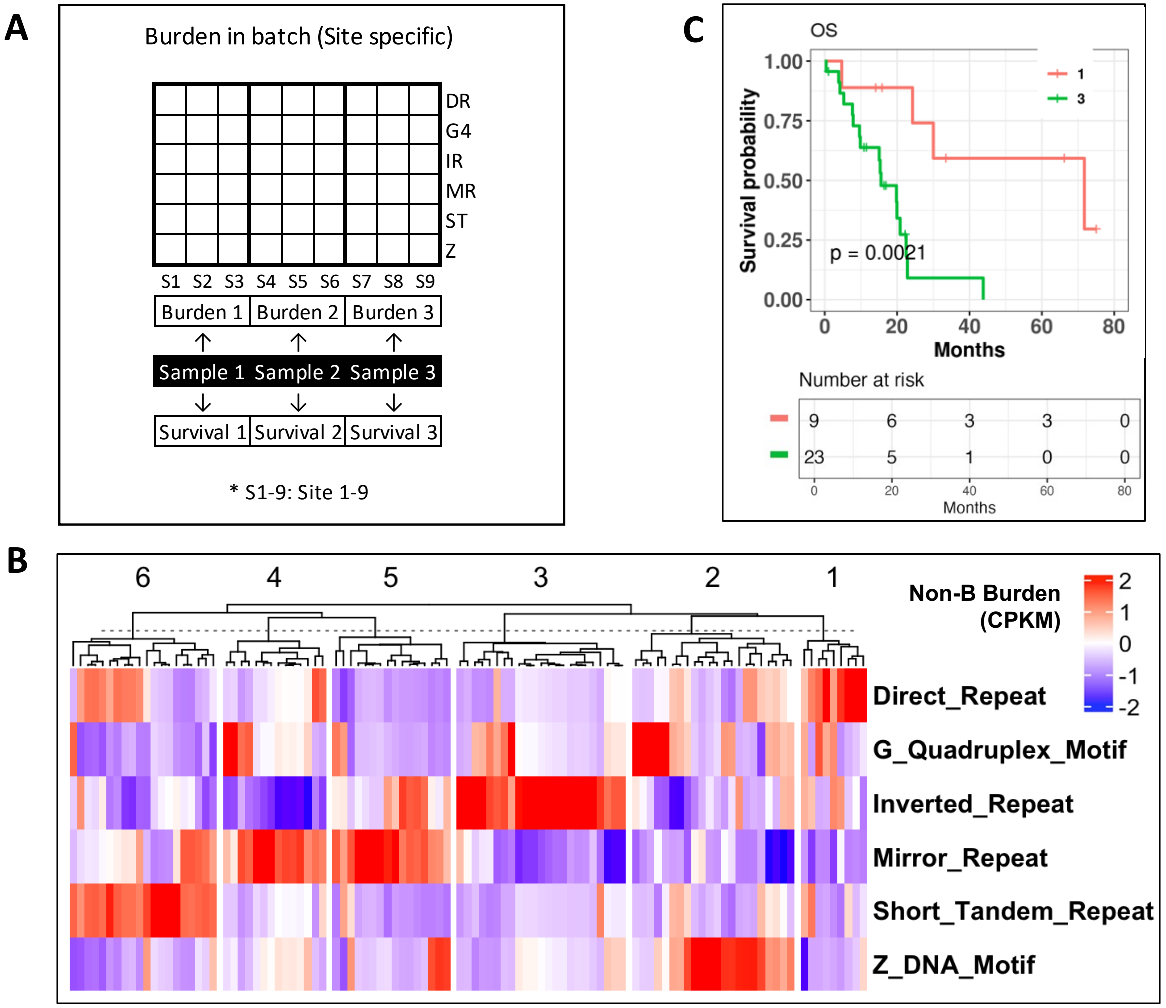

Fig S4. Case 3: Mutation-localized non-B burdens in multiple samples(sample-level and site-specific).
(A) Graphical summary of non-B burden calculation at the sample level. (B) Heatmap of 104 early-stage pancreatic cancer samples from TCGA-PAAD with mutation and survival data clustering of mutation site-specific, sample-level non-B burdens. (C) Cluster 1 (DR high) and Cluster 3 (IR high) show a significant overall survival (OS) difference (p = 0.0021).
